# A Probabilistic Decision-Making Scoring System for Quality and Safety Management in *Aloreña de Málaga* Table Olive Processing

**DOI:** 10.3389/fmicb.2017.02326

**Published:** 2017-11-29

**Authors:** Miguel Á. Ruiz Bellido, Antonio Valero, Eduardo Medina Pradas, Verónica Romero Gil, Francisco Rodríguez-Gómez, Guiomar D. Posada-Izquierdo, Francisco Rincón, Aricia Possas, Rosa M. García-Gimeno, Francisco N. Arroyo-López

**Affiliations:** ^1^Regulatory Council of PDO Aloreña de Málaga Table Olives, Malaga, Spain; ^2^Department of Food Science and Technology, Universidad de Córdoba, Córdoba, Spain; ^3^Food Biotechnology Department, Instituto de la Grasa (Consejo Superior de Investigaciones Científicas), University Campus Pablo de Olavide, Seville, Spain

**Keywords:** table olives, HACCP, decision-support system, performance hygiene and safety scores, sensitivity analysis

## Abstract

Table olives are one of the most representatives and consumed fermented vegetables in Mediterranean countries. However, there is an evident lack of standardization of production processes and HACCP systems thus implying the need of establishing decision-making tools allowing their commercialization and shelf-life extension. The present work aims at developing a decision-making scoring system by means of a probabilistic assessment to standardize production process of *Aloreña de Málaga* table olives based on the identification of potential hazards or deficiencies in hygienic processes for the subsequent implementation of corrective measures. A total of 658 microbiological and physico-chemical data were collected over three consecutive olive campaigns (2014–2016) to measure the variability and relative importance of each elaboration step on total hygienic quality and product safety. Three representative companies were visited to collect samples from food-contact surfaces, olive fruits, brines, air environment, olive dressings, water tanks, and finished/packaged products. A probabilistic assessment was done based on the establishment of Performance Hygiene and Safety Scores (PHSS 0–100%) through a standardized system for evaluating product acceptability. The mean value of the global PHSS for the *Aloreña de Málaga* table olives processing (PHHS_FTOT_) was 64.82% (90th CI: 52.78–76.39%) indicating the high variability among facilities in the evaluated processing steps on final product quality and safety. Washing and cracking, and selection and addition of olive dressings were detected as the most deficient ones in relation to PHSS_Fi_ values (*p* < 0.05) (mean = 53.02 and 56.62%, respectively). The relative contribution of each processing step was quantified by different experts (*n* = 25) from the *Aloreña de Málaga* table olive sector through a weighted PHSS (PHSS_w_). The mean value of PHSS_w_ was 65.53% (90th CI: 53.12–77.52%). The final processing steps obtained higher values for PHSS_w_ being the finished product the most relevant one (mean = 18.44%; 90% CI: 10.34–25.33%). Sensitivity analysis concluded that intervention measures focused on reducing the contamination of washing brines could lead to an improvement of PHSS_FTOT_ value to 67.03%. The present work can be potentially applied in the *Aloreña de Málaga* table olive food sector for improving food quality and safety assurance.

## Introduction

Table olives are one of the most representative and consumed fermented vegetables in Mediterranean countries (Garrido-Fernandez et al., 1997; Arroyo-López et al., 2012, 2016). According to the recent statistics provided by the International Olive Oil Council (IOOC), European production has raised in 2015/16 to 859.8 mT whereas consumption also showed an increasing trend to 410.7 mT (IOOC, 2017). The global consumption of table olives in recent years has multiplied by 2.7, increasing by 182.0% over the period 1990/91–2016/17. Spain was ranked as the main producer in the world as well as the main consumer with 4.1 kg/person/year.

In the last years, consumers are demanding healthier and more convenient table olive preparations based on traditional processes. In Spain, *Aloreña de Málaga* green table olive has a Protected Designation of Origin (PDO) due to their peculiar characteristics of elaboration and geographical production region (Guadalhorce Valley, Málaga, Spain). Due to its low-to-moderate concentrations of oleuropein, the processing does not include alkaline debittering. Thus, they are produced as directly brined cracked green olives and seasoned with diverse herbs and species before packaging (López-López and Garrido-Fernández, 2006). Their differential characteristics regarding other table olive varieties limits the possibility of applying a heat treatment sufficiently high to destroy or reduce the microbial load in the packaged product. This requires the implementation of alternative preservation processes to allow increasing the shelf-life and further commercialization of finished products.

The microbiological safety of foods is managed by the effective implementation of control measures within a Food Quality Safety Management Systems (FQSMS) including prerequisite programme (PRP) and hazard analysis and critical control points (HACCP) that have been validated, where appropriate, throughout the food chain to minimize contamination and improve food safety (Valero et al., 2017). An integrated approach to food safety covers all sectors of the food chain (Regulation EC 178/2002, Commission Regulation, 2002) in response to requirements demanded by customers, competent authorities and certification bodies. Hygienic requirements for foodstuffs (Regulation EC 852/2004, Commission Regulation, 2004) implemented in the EU have urged the need to develop more sophisticated food quality and safety assurance standards and guidelines (Tzamalis et al., 2016). For the table olives sector, the codex standard (CODEX STAN 66-1981, review 1987 and 2013, Codex Alimentarius Commission, 1981) and the Trade Standard Applying to Table Olives (IOC, 2004) recommend that the product covered by these documents must be prepared and handled in accordance with the appropriate sections of the General Principles of Food Hygiene (CAC/RCP 1-1969; Codex Alimentarius Commission, 1969b), the Code of Hygienic Practice for Low-Acid and Acidified Low-Acid Canned Foods (CAC/RCP 23-1979, Codex Alimentarius Commission, 1969a, 1979), and the Code of Hygienic Practice for Canned Fruit and Vegetable Products (CAC/RCP 2-1969, Codex Alimentarius Commission, 1969a). In addition, the product should comply with any microbiological criteria established in accordance with the Principles for the Establishment and Application of Microbiological Criteria for Foods (CAC/GL 21-1997, Codex Alimentarius Commission, 1997; Regulation (EC) 1441/2007, Commission Regulation, 2007).

The development of a FQSMS requires quantitative tools able to assess the acceptance of final products. However, there is an evident lack of standardization in the table olive sector, thus implying the need of establishing decision-making tools allowing their commercialization and shelf-life extension. Lack of experience, knowledge and human and financial resources make difficult the implementation of standardized FQSMS in industry (Tzamalis et al., 2016). Further, production of table olives as fermented products could not be standardized since several factors such as variations in olive composition according to the season, spontaneous fermentation processes, limited technological capabilities in the company or lack of scientific and technical knowledge by industry's operators. Specifically, the manufacturing process of *Aloreña de Málaga* table olives is carried out by small and medium enterprises placed in, or very close to, the region of production. This fact together with the limited shelf-life of final products due to the presence of high residual sugars, spoilage microorganism, clouding or brines and swelling containers, make the distribution area very limited and do not allow in some cases exportation to other countries (Romero-Gil et al., 2016). There are previous studies dealing with the development of FQSMS in other food commodities demonstrating their usefulness to improve food quality and safety. One of the best examples is the Food Safety Management System- diagnostic instrument (FSMS-DI), which contributes to the measurement of the performance of the FSMS in an organization suggested for edible oil or fresh produce chains (Nanyunja et al., 2015; Ren et al., 2016). Further, development of scoring systems (Stadlmüller et al., 2017) and best practice scores (Tzamalis et al., 2016) for the assessment of FQSMS are also reported. However, these systems are deterministic approaches mainly based on performance of questionnaires or microbial data on targeted hazards, being not potentially applied to the table olive sector. The establishment of risk quality or safety margins by food operators is desirable since quantification of variability associated to products and processes can be quantified. Furthermore, these measures are in line with the preventive Food Safety Modernization Act approach implemented in US (Grover et al., 2016).

In this study, a probabilistic approach is suggested to assess the performance of quality and safety of *Aloreña de Málaga* table olives production. Based on physico-chemical and microbiological data collected from three representative companies and seasons in Southern Spain, a decision-scoring system was developed establishing Performance Hygiene and Safety Scores (PHSS) to identify potential factors and processing steps to operationalize hygiene and safety of table olive processing.

## Materials and methods

### Study design and facilities

**Figure 1:**
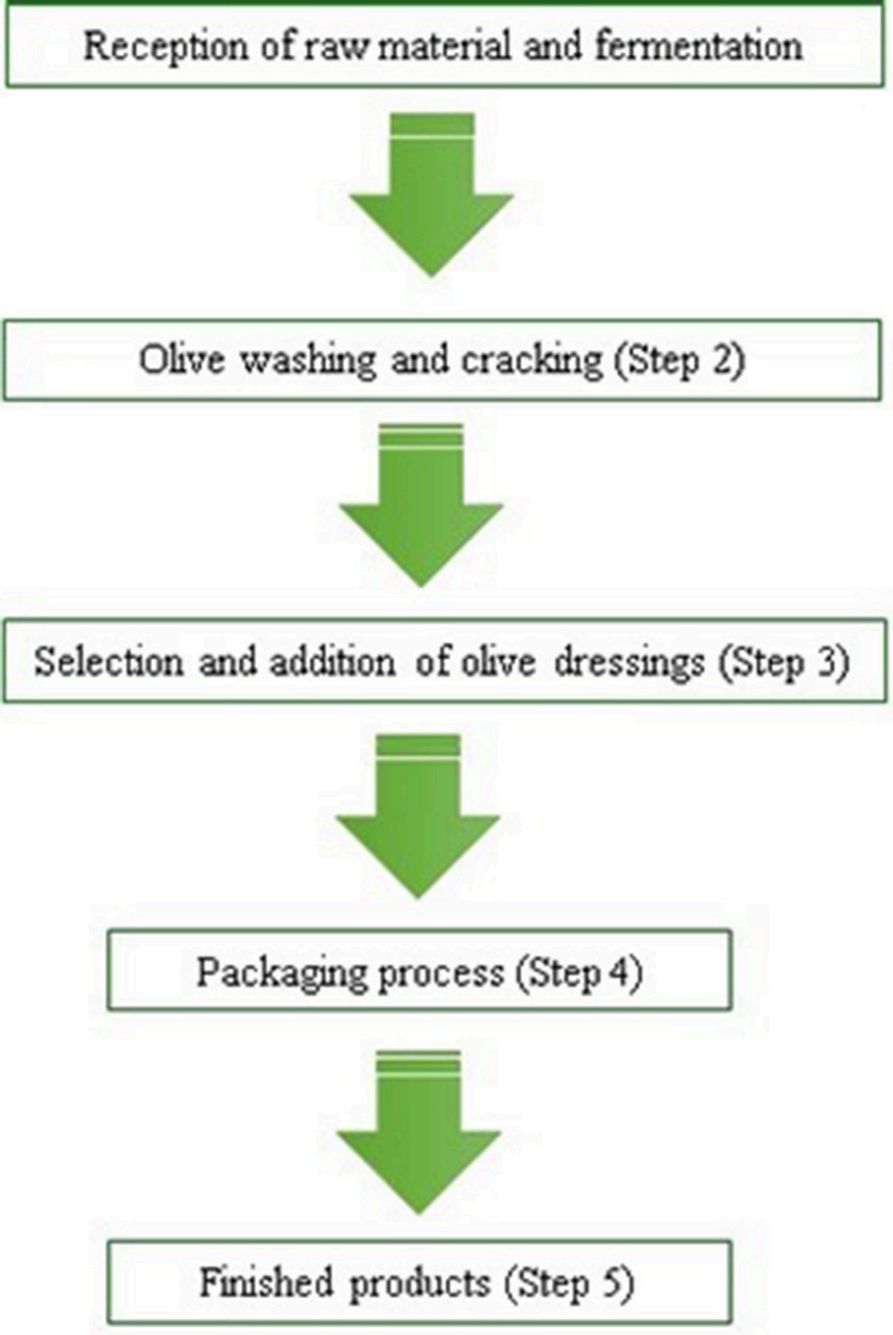
Flow diagram of the elaboration process of *Aloreña* table olives shared by the different industries visited in the study.

This study was performed in three different small and medium companies dedicated to table olives production located in Southern Spain (Valle del Guadalhorce, Málaga, Spain) which process all *Aloreña de Málaga* table olives. The experimental work was conducted in three consecutive campaigns from 2014 to 2016. Types of samples, processing steps to analyse and sampling planning were previously agreed with the quality inspector of each company as indicated in their different industry's Self-Control Plan (HACCP). The processing steps considered for the present study were based on the traditional elaboration of *Aloreña* table olives and they are shown in Figure 1. From each step, different microbiological and physico-chemical analyses were performed as described below to determine their influence on final product quality, hygiene and safety.

### Microbiological analyses

#### Samples collection

According with the sampling planning (Tables 1–5), different types of samples were collected in the industry, transferred to sterile containers, transported to the laboratory at refrigeration (2–4°C) conditions and analyzed within 24 h after collection.

**Table 1:** Analyses performed, parameters, concentration and scores obtained from samples collected at processing step No. 1 (reception of raw materials and fermentation).

**Type of sample (No. analyses)**	**Units**	Parameter^*^	**Mean concentration (95% CI)**	**Score (mean, 95% CI)**
Air environment (16)	cfu/m^3^	MB	208.87 (181.44, 236.31)	1.56 (1.95, 2.30)
		Y/M	56.50 (24.98, 88.02)	1.00 (0.74, 1.26)
Olive brine (32)	log_10_ cfu/ml	MB	6.20 (5.91, 6.37)	2.25 (1.77, 2.73)
		Y/M	5.00 (4.61, 5.21)	1.75 (1.35, 2.15)
		LAB	6.30 (5.98, 6.48)	2.00 (1.55, 2.45)
		Ent	<1.30 (–)	0.00 (–)^**^
Olive fruit (48)	log_10_ cfu/g	MB	5.88 (5.54, 6.06)	1.75 (1.42, 2.08)
		Y/M	4.30 (4.04, 4.47)	1.25 (0.96, 1.54)
		LAB	5.79 (5.48, 5.97)	1.50 (1.16, 1.84)
		Ent	<1.30 (–)	0.00 (–)
		CPS	<1 (–)	0.00 (–)
		SRC	1.07 (1.01, 1.11)	0.50 (0.20, 0.80)
Olive brine (12)	–	pH	4.23 (4.04, 4.42)	1.50 (0.52, 2.48)
	g/100 ml	FA	0.77 (0.62, 0.92)	0.00 (–)
	% (w/v)	NaCl	7.44 (7.10, 7.77)	0.00 (–)
Water (18)	cfu/100 ml	MB	14.30 (<10, 23.96)	0.83 (0.64, 1.02)
		Col	<10 (–)	1.00 (0.28, 1.72)
		SRC	<10 (–)	1.00 (0.28, 1.72)

* *MB, Mesophilic bacteria; Y/M, yeast/molds; LAB, Lactic-acid bacteria; Ent, Enterobacteriaceae; CPS, coagulase positive Staphylococci; SRC, Sulphite Reducing Clostridia; FA, free acidity; Col, total coliforms*.

** *CI 95% could not be estimated*.

**Table 2:** Analyses performed, parameters, concentration, and scores obtained from samples collected at processing step No. 2 (washing and cracking).

**Type of sample (No. analyses)**	**Units**	Parameter^*^	**Mean concentration (95% CI)**	**Score (mean, 95% CI)**
Air environment (16)	cfu/m^3^	MB	210 (183.47, 236.77)	2.00 (–)
		Y/M	103 (79.83, 126.91)	1.38 (1.14, 1.61)
Hopper surface (16)	cfu/cm^2^	MB	36.60 (5.44, 67.70)	2.63 (2.14, 3.00)
		Ent	73.5 (12.80, 134.17)	1.50 (0.76, 2.24)
Olive fruit (36)	log_10_ cfu/g	MB	5.70 (5.41, 5.87)	1.67 (1.22, 2.11)
		Y/M	4.66 (4.40, 4.82)	0.67 (0.33, 1.00)
		LAB	5.95 (5.76, 6.08)	1.67 (1.22, 2.11)
		Ent	<1.30 (–)^**^	0.00 (–)
		CPS	1.07 (<1, 1.11)	0.17 (0.03, 0.30)
		SRC	<1.30 (–)	0.00 (–)
Olive brine (24)	log_10_ cfu/ml	MB	5.04 (4.59, 5.25)	1.83 (1.37, 2.30)
		Y/M	4.87 (4.63, 5.03)	1.83 (1.37, 2.30)
		LAB	6.59 (6.03, 6.83)	1.33 (0.73, 1.94)
		Ent	4.38 (3.95, 4.59)	1.33 (0.79, 1.88)
Water (18)	cfu/100 ml	MB	1.34 (1.06, 1.50)	0.83 (0.64, 1.02)
		Col	<1.30 (–)	1.00 (0.28, 1.71)
		SRC	<1.30 (–)	1.00 (0.28, 1.71)

* *MB, Mesophilic bacteria; Y/M, yeast/molds; LAB, Lactic-acid bacteria; Ent, Enterobacteriaceae; CPS, coagulase positive Staphylococci; SRC, Sulphite Reducing Clostridia; Col, total coliforms*.

** *CI 95% could not be estimated*.

**Table 3:** Analyses performed, parameters, concentration, and scores obtained from samples collected at processing step No. 3 (selection and addition of olive dressings).

**Type of sample (No. analyses)**	**Units**	Parameter^*^	**Mean concentration (95% CI)**	**Score (mean, 95% CI)**
Air environment (16)	cfu/m^3^	MB	182 (150.76, 213.80)	2.25 (2.02, 2.48)
		Y/M	81.4 (64.62, 98.12)	1.25 (1.02, 1.48)
Conveyor belt (12)	cfu/cm^2^	MB	18,000 (0, 23,567)	3.00 (–)^**^
		Ent	94.2 (22.21, 166.08)	1.33 (0.60, 2.07)
Olive fruit (36)	log_10_ cfu/g	MB	5.19 (5.06, 5.30)	1.50 (1.23, 1.77)
		Y/M	4.07 (3.76, 4.25)	1.00 (0.71, 1.29)
		LAB	4.93 (4.70, 5.08)	1.33 (1.00, 1.67)
		Ent	2.30 (–)	0.50 (0.10, 0.90)
		CPS	<1.30 (–)	0.17 (0.03, 0.30)
		SRC	<1.30 (–)	0.00 (–)
Olive dressing: red pepper (36)	log_10_ cfu/g	MB	3.21 (3.09, 3.30)	0.33 (0.16, 0.50)
		Y/M	3.37 (2.93, 3.59)	0.50 (0.23, 0.77)
		LAB	2.72 (2.02, 2.98)	0.17 (0.03, 0.30)
		Ent	1.70 (1.00, 1.95)	0.50 (0.10, 0.90)
		CPS	2.90 (2.63, 3.07)	1.50 (0.96, 2.04)
		SRC	<1.30 (–)	0.00 (–)
Olive dressing: garlic (36)	log_10_ cfu/g	MB	4.61 (4.26, 4.79)	0.83 (0.40, 1.27)
		Y/M	3.13 (2.80, 3.31)	0.33 (0.16, 0.50)
		LAB	3.80 (3.50, 3.98)	0.83 (0.51, 1.15)
		Ent	3.15 (2.60, 3.38)	1.00 (0.49, 1.50)
		CPS	2.50 (2.16, 2.69)	1.00 (0.49, 1.50)
		SRC	<1.30 (–)	0.00 (–)
Olive dressing: herbs (36)	log_10_ cfu/g	MB	7.34 (6.69, 7.59)	2.50 (2.23, 2.77)
		Y/M	5.72 (5.43, 5.90)	1.67 (1.27, 2.06)
		LAB	5.56 (5.09, 5.78)	1.00 (0.49, 1.50)
		Ent	6.45 (5.75, 6.71)	2.00 (1.50, 2.50)
		CPS	4.42 (3.85, 4.66)	2.50 (2.10, 2.90)
		SRC	2.03 (1.74, 2.21)	1.00 (0.71, 1.29)
Handlers' gloves (12)	cfu/cm^2^	MB	2.06 (0.80, 2.35)	1.60 (1.09, 2.11)
		Ent	<1 (–)	0.00 (–)

* *MB, Mesophilic bacteria; Y/M, yeast/molds; LAB, Lactic-acid bacteria; Ent, Enterobacteriaceae; CPS, coagulase positive Staphylococci; SRC, Sulphite Reducing Clostridia; FA, free acidity; Col, total coliforms*.

** *CI 95% could not be estimated*.

**Table 4:** Analyses performed, parameters, concentration, and scores obtained from samples collected at processing step No. 4 (packaging processes).

**Type of sample (No. analyses)**	**Units**	Parameter^*^	**Mean concentration (95% CI)**	**Score (mean, 95% CI)**
Air environment (16)	cfu/m^3^	MB	184 (150.46, 216.73)	2.17 (1.97, 2.37)
		Y/M	84.2 (63.77, 104.56)	1.33 (1.08, 1.59)
Packaging containers (12)	cfu/cm^2^	MB	<1 (–)^**^	0.50 (0.00, 1.19)
		Ent	<1 (–)	0.00 (–)
Olive fruit (36)	log_10_ cfu/g	MB	4.39 (4.08, 4.58)	1.33 (1.07, 1.60)
		Y/M	3.80 (3.57, 3.95)	1.00 (0.71, 1.29)
		LAB	3.75 (3.11, 3.99)	0.50 (0.23, 0.77)
		Ent	2.68 (2.18, 2.91)	1.50 (0.96, 2.03)
		CPS	<1.30 (–)	0.33 (0.16, 0.50)
		SRC	<1.30 (–)	1.00 (0.49, 1.51)
Olive brine (24)	log_10_ cfu/ml	MB	1.62 (<1.30, 1.84)	0.17 (0.00, 0.33)
		Y/M	<1.30 (–)	0.00 (–)
		LAB	<1.30 (–)	0.00 (–)
		Ent	<1.30 (–)	0.00 (–)
Handlers gloves (12)	cfu/cm^2^	MB	2.04 (<1.30, 2.34)	1.40 (0.75, 2.05)
		Ent	<1 (–)	0.60 (0.00, 1.35)
Water (18)	cfu/100 ml	MB	14.30 (<10, 23.96)	0.83 (0.64, 1.02)
		Col	<10 (–)	0.00 (–)
		SRC	<10 (–)	0.00 (–)

* *MB, Mesophilic bacteria; Y/M, yeast/molds; LAB, Lactic-acid bacteria; Ent, Enterobacteriaceae; CPS, coagulase positive Staphylococci; SRC, Sulphite Reducing Clostridia; Col, total coliforms*.

** *CI 95% could not be estimated*.

**Table 5:** Analyses performed, parameters, concentration, and scores obtained from samples collected at processing step No. 5 (finished product).

**Type of sample (No. analyses)**	**Units**	Parameter^*^	**Mean concentration (95% CI)**	**Score (mean, 95% CI)**
Olive brine (48)	log_10_ cfu/ml	MB	3.08 (3.00, 3.14)	1.67 (1.52, 1.81)
		Y/M	3.20 (3.06, 3.31)	1.33 (1.10, 1.56)
		LAB	3.82 (3.48, 4.01)	1.33 (1.04, 1.62)
		Ent	<1.30 (–)^**^	0.17 (0.05, 0.28)
		CPS	<1.30 (–)	0.00 (–)
		SRC	<1.30 (–)	0.50 (0.15, 0.85)
Olive fruit (48)	log_10_ cfu/ml	MB	3.89 (3.65, 4.05)	0.83 (0.56, 1.11)
		Y/M	3.85 (3.70, 3.96)	1.33 (1.19, 1.48)
		LAB	4.35 (4.09, 4.52)	1.00 (0.75, 1.25)
		Ent	<1.30 (–)	0.17 (0.05, 0.28)
		CPS	<1.30 (–)	0.50 (0.35, 0.65)
		SRC	<1.30 (–)	0.50 (0.15, 0.85)
		LM	<−1.40 (–)	0.00 (–)
		Salm	<−1.40 (–)	0.00 (–)
Olive brine (24)	–	pH	4.23 (4.14, 4.32)	1.50 (1.11, 1.89)
	mEq/ml	FA	0.31 (0.28, 0.34)	0.50 (0.26, 0.74)
	% (w/v)	NaCl	5.44 (5.36, 5.52)	1.00 (0.75, 1.25)
	g/l	Sugar	2.70 (2.00, 3.40)	0.50 (0.35, 0.65)

* *MB, Mesophilic bacteria; Y/M, yeast/molds; LAB, Lactic-acid bacteria; Ent, Enterobacteriaceae, CPS, coagulase positive Staphylococci; SRC, Sulphite Reducing Clostridia; LM, L. monocytogenes; Salm, Salmonella sp.; Col, total coliforms*.

** *CI 95% could not be estimated*.

#### Enumeration of microbial populations in the different types of samples

To enumerate microbial populations in brines, samples (10 ml), if necessary, were serially diluted in sterile saline solution (0.9% NaCl) and plated (50 μl) in the correspondent culture media described below through using a Spiral Plater model dwScientific (Don Whitley Scientific Limited, UK). After incubation periods at the different temperatures according to the microbial group analyzed in this type of sample [lactic acid bacteria (LAB), yeasts and molds (Y/M), mesophilic bacteria (MB), and *Enterobacteriaceae* (Ent)], colonies were counted by using an Image Analysis System model CounterMat v.3.10 (IUL, Barcelona, Spain). Results were expressed at log_10_ cfu/ml.

For determination of microorganisms present in olive fruits [MB, Y/M, LAB, Ent, coagulase positive *Staphylococci* (CPS), sulphite reducing clostridia (SRC), *Listeria monocytogenes* (LM), and *Salmonella* sp. (Salm)], two olives (approximately 10 g) were washed with sterile saline solution (0.85% v/v) and deboned at sterile conditions. Then, a decimal dilution of fruit flesh in saline solution (90 ml) was homogenized in a Stomacher 400 Circulator Blender (Seward Laboratory System, UK) for 5 min. Afterwards, 50 μl were plated in the selective culture media. Results were expressed at log_10_ cfu/g.

To enumerate the number of microorganisms present in olive dressings (MB, Y/M, LAB, Ent, CPS, and SRC), 10 g of each seasoning material (garlic, red pepper and herbal mixture) were singly homogenized for 5 min in Stomacher with 90 ml of buffered peptone water (0.1%). Afterwards, 50 μl of the solutions were plated in the different culture media. Results were expressed at log_10_ cfu/g.

To determine the presence of microorganisms in water, samples (1,000 ml) were poured into sterile flasks and re-suspended with 5% solution of sodium thiosulfate (Panreac, Barcelona, Spain) to remove the residual effect of free chlorine. Then, samples were filter-sterilized using 0.22 μm diameter filters (Merck Millipore, Massachusetts, US). Then, filters were transferred to different selective media for analysis of the microbial groups specified in the Spanish Royal Decree (RD 140/2003, Royal Decree, 2003) such as MB, coliforms (Col), and SRC. Results were expressed at log_10_ cfu/ml.

Microbial air quality was determined by using an Air Sampler (SAS Super 180TM, Scharlab, Barcelona, Spain) searching for MB and Y/M as microbial indicators. The volume of air was fixed at 500 litters. Probable counts (*Pr*, cfu/m^3^, statistical probability of multiple particles passing through the same hole) were obtained using a conversion table provided by the manufacturer.

The analysis of food-contact surfaces was carried out using MB and Ent as microbial indicators. Sterile polypropylene swabs (Nuovo Aptaca, Canelli, Italy) with amies medium were used for surface sampling. Each surface was swabbed using a 10 × 10 cm sterile metal template, then the swab head (1–2 cm) was aseptically cut and immersed in 3 ml test tubes of 0.1% buffered peptone water. In the case of handlers' gloves samples, the inner part was swabbed and the area in contact with hands was estimated as 225.07 ± 21.07 cm^2^ for men and 188.03 ± 16.08 cm^2^ for women (Ren et al., 2010). Results were expressed at cfu/cm^2^.

Selective culture media used for enumeration of LAB was to DeMan Rogosa and Sharpe (MRS) agar (Oxoid, Basingstoke, UK) supplemented with 0.02% of sodium azide (Sigma, St. Louis, US) following by an incubation at 37°C for 48 h. Y/M were enumerated with the Yeast Mold agar (YM, Disco, Becton y Dickinson Company, Spark, MD, US) supplemented with 0.005% of gentamycin and oxy-tetracycline sulfate (Oxoid). Samples were incubated at 30°C for 48 h. Ent were counted using Violet Crystal Red Bile Dextrose (VRBD) agar (Merck, Darmstadt, Germany) after an incubation at 37°C for 24 h. MB were enumerated with Plate Count Agar (PCA, Oxoid) after an incubation at 28°C for 24h. CPS were enumerated following the ISO method (ISO: 6888-2: ISO, 1999) in Baird Parker supplemented with fibrinogen and rabbit plasmid (incubation at 37°C for 24 h). SRC were counted using Tryptose Sulphite Cycloserine (TSC) agar (Oxoid) after an incubation at 37°C for 24 h in anaerobic jars. Finally, presence of LM and Salm was confirmed using the ISO methods [ISO 11290-1/-2 (ISO, 2004, 2017) for LM and ISO 6579 (ISO, 2002) for Salm, respectively].

### Physico-chemical analyses

The analyses of the olive brine for pH, salt, and titratable/free acidity (FA) were carried out using the routine methods described by Garrido-Fernandez et al. (1997). Total sugar content in brine (g/l) was determined by HPLC according to the methods developed by Sánchez et al. (2000) by the summation of values obtained for glucose, fructose, sucrose and mannitol.

### Development of a decision-making scoring system to operationalize hygiene and safety of table olive processing

#### Scoring system

In this study, a quantitative system assessing the food hygiene and safety throughout the elaboration process of table olives was established. This was done through a scoring system assigning different weighted values to microbiological and physico-chemical results obtained at the different steps in the elaboration chain (Figure 1). Scores ranged from 0 to 3, indicating the best and worst quality/safety conditions, respectively. Assigned scores and ranges are represented in Table 6. which describes how concentrations are translated to scores and the references used. These values were based on previous published studies, Codex standards for table olives, national and European legislations regarding different food criteria applied to samples and parameters evaluated (Al Dagal et al., 1992; Federation des Industries Condimentaires de France, 2000; Royal Decree 1230/2001, Royal Decree, 2001; Royal Decree 140/2003, Royal Decree, 2003; IOC, 2004; Sneed et al., 2004; Regulation EC 1441/2007, Commission Regulation, 2007; Codex 66-1981, 2013, Codex Alimentarius Commission, 1981).

**Table 6:** Scoring system assigned to the different physico-chemical and microbiological parameters analyzed and samples collected.

**Type of sample**	Parameters^*^	**Units**	**Scores**				Source^**^
Air environment	MB and Y/M	cfu/m^3^	<10 (0)	10–100 (1)	101–300 (2)	>300 (3)	1
Food-contact surfaces	MB	cfu/cm^2^	<1 (0)	1–10 (1)	11–100 (2)	>100 (3)	2
	Ent	cfu/cm^2^	<1 (0)	1–5 (1)	5–10 (2)	>10 (3)	2
Olive fruits (semi-elaborated)	MB	cfu/g	<10^3^ (0)	10^3^–10^4^ (1)	10^4^−10^6^ (2)	>10^6^ (3)	3, 4, 5
	Ent	cfu/g	< 20	21–50	51–100	>100	3, 4, 5
	LAB	cfu/g	<10^3^ (0)	10^3^–10^4^ (1)	10^4^–10^6^ (2)	>10^6^ (3)	3, 4, 5
	Y/M	cfu/g	<10^3^ (0)	10^3^–10^4^ (1)	10^4^–10^5^ (2)	>10^5^ (3)	3, 4, 5
	CPS	cfu/g	<20 (0)	21–50	51–100	>100	3, 4, 5
	SRC	cfu/g	<20 (0)	–	–	≥20 (3)	3, 4, 5
Olive fruits (finished product) and olive dressings (garlic and red pepper)	MB	cfu/g	<10^3^ (0)	10^3^−10^4^ (1)	10^4^–10^5^ (2)	>10^5^ (3)	3, 4, 5
	Ent	cfu/g	<20	21–50	51–100	>100	3, 4, 5
	LAB	cfu/g	<10^2^ (0)	10^2^–10^4^ (1)	10^4^–10^5^ (2)	>10^5^ (3)	3, 4, 5
	Y/M	cfu/g	<10^2^ (0)	10^2^–10^4^ (1)	10^4^–10^5^ (2)	>10^5^ (3)	3, 4, 5
	CPS	cfu/g	<20 (0)	21–50	51–100	>100	3, 4, 5
	SRC	cfu/g	<20 (0)	–	–	≥20 (3)	3, 4, 5
	LM	cfu/g	<1 /25g (0)	–	–	≥1 /25 g (3)	6
	Salm	cfu/g	<1 /25g (0)	–	–	≥1 /25 g (3)	6
Brines	MB	cfu/ml	<10^2^ (0)	10^2^−10^3^ (1)	10^3^−10^5^ (2)	>10^5^ (3)	3, 4, 5
	Ent	cfu/ml	<20	21–50	51–100	>100	3, 4, 5
	LAB	cfu/ml	<10^2^ (0)	10^2^–10^3^ (1)	10^3^–10^5^ (2)	>10^5^ (3)	3, 4, 5
	Y/M	cfu/ml	<10^2^ (0)	10^2^–10^3^ (1)	10^3^–10^5^ (2)	>10^5^ (3)	3, 4, 5
	pH	–	<4.0 (0)	4.0–4.2 (1)	4.2–4.3 (2)	>4.3 (3)	7
	FA	g/100 ml	>0.3 (0)	0.2–0.3 (1)	0.1–0.2 (2)	<0.1 (3)	7
	NaCl	% (w/v)	>6.0 (0)	5.5–6.0 (1)	5.0–5.5 (2)	<5.5 (3)	7
	Sugar	% (g/l)	<2.0 (0)	2.0–9.0 (1)	9.0–19.0 (2)	>19.0 (3)	7
Olive dressing (herbs)	SRC	cfu/g	<20	21–100	101–10^3^	>10^3^	3, 4, 5
Water	MB	cfu/100 ml	<1 (0)	1–50 (1)	51–100 (2)	>100 (3)	8
	Col	cfu/100 ml	<1 (0)	–	–	≥1 (3)	8
	SRC	cfu/100 ml	<1 (0)	–	–	≥1 (3)	8

* *MB, Mesophilic bacteria; Y/M, yeast/molds; LAB, Lactic-acid bacteria; Ent, Enterobacteriaceae; CPS, coagulase positive Staphylococci; SRC, Sulphite Reducing Clostridia; FA, free acidity; Col, total coliforms; LM, L. monocytogenes; Salm, Salmonella sp*.

** *1 (Al Dagal et al., 1992); 2 (Sneed et al., 2004); 3 [Codex Standard for Table Olives (Codex 66-1981, 2013, Codex Alimentarius Commission, 1981)]; 4 (Trade Standard Applying to Table Olives, IOC, 2004); 5 [Code des Bonnes Pratiques Loyales pour les Olives de Table (Federation des Industries Condimentaires de France, 2000)]; 6 [Commission Regulation (EC) No 1441/2007 of 5 December 2007 amending Regulation (EC) No 2073/2005 on microbiological criteria for foodstuffs, Commission Regulation, 2007); 7 (Royal Decree 1230/2001, of 8 November, approving the Technical-sanitary Regulation for the elaboration, distribution and sale of table olives); 8 [Royal Decree 140/2003 (Royal Decree, 2003) of 7 February, establishing the sanitary quality criteria of water for human consumption]*.

#### Calculation of performance hygiene and safety scores

The obtained results were processed and the correspondent scores assigned to each analytical data in accordance to the criteria represented in Table 6. Then, a probabilistic model was created in @Risk v7.5 (Palisade Corporation) to quantify variability associated to the elaboration process and to identify potential steps and factors that could influence on the final degree of hygiene and safety.

The variables used for model development were:

– *F*_*i*_ defining the processing step *i* (Figure 1) (*i* ranges from 1 to 5),
– *T*_*i*_ is the type of sample collected within the *i*th processing step (i.e., air environment, olive fruits, brines, etc.)
– *P*_*i*_ is the parameter analyzed corresponding to *T*_*i*_ within the processing step *F*_*i*_ (i.e., MB, LAB, pH, etc.) and,
– *S*_*i*_ is the assigned score to the *i*th parameter, ranging from 0 to 3.

As an example, for the processing step *F*_1_ (reception of raw material and storage), and type of sample *T*_1_ (air environment) the assigned scores as 0, 1, 2, and 3 were summed up for all parameters as follows:

(1) $$\begin{array}{l} \left(F_{1},T_{1}\right)=\Sigma S_{0}\left(P_{i}\right);\Sigma S_{1}\left(P_{i}\right);\Sigma S_{2}\left(P_{i}\right);\Sigma S_{3}\left(P_{i}\right); \end{array}$$

being *P*_*i*_ the number of parameters evaluated for this type of sample (in this case, MB and Y/M).

Let N_0_ to N_3_ be the number of times the scores were assigned as 0, 1, 2, and 3, then: *N*_0_ = Σ*S*_0_; *N*_1_ = ΣS_1_; *N*_2_ = Σ*S*_2_; *N*_3_ = ΣS_3_. Once *N*_0_ to *N*_3_ values were obtained, within each processing step (*F*) and type of sample (*T*), the correspondent probabilities (*p*) associated to each score were calculated as:

(2) $$\begin{array}{l} p_{0}=\frac{N_{0}}{N_{0}+N_{1}+N_{2}+N_{3}};\;p_{1}=\frac{N_{1}}{N_{0}+N_{1}+N_{2}+N_{3}};\\ p_{2}=\frac{N_{2}}{N_{0}+N_{1}+N_{2}+N_{3}};\;p_{3}=\frac{N_{3}}{N_{0}+N_{1}+N_{2}+N_{3}} \end{array}$$

being *p*_0_ + *p*_1_ + *p*_2_ + *p*_3_ = 1. A discrete function was implemented in @Risk to assign any possible values from 0 to 3 as a function of the calculated probabilities (*p*). The resulting values from the discrete distribution (*D*_1_, *D*_2_ … *D*_*i*_) corresponding to the types of samples and parameters evaluated were summed up to obtain the score for the ith processing step (*D*_*Fi*_), defined as:

(3) $$\begin{array}{l} D_{Fi}=D_{1}T_{1}+D_{2}T_{2}+\ldots +D_{i}T_{i} \end{array}$$

To measure the degree of fulfillment of the ith processing step on product quality and safety, a Performance Hygiene and Safety Score (PHSS_Fi_, %) was obtained. PHHS_Fi_ values ranged from 0 to 100% indicating the percentage of fulfillment of F_i_ on the overall quality and safety of the process and finished product and was calculated as follows:

(4) $$\begin{array}{l} PHSS_{Fi}=1-\left(\frac{D_{Fi}}{D_{maxFi}}\right)x\;100 \end{array}$$

where *D*_*maxFi*_ was defined as the maximum score that can be potentially obtained for the processing step *F*_*i*_ (being representative of the worst-case scenario):

$$\begin{array}{l} D_{maxFi}=3\times \text{number of parameters evaluated in }F_{i}. \end{array}$$

This worst-case scenario was considered as the score 3 was associated to the poorest hygienic conditions. This measure was needed for model development to relativize the PHSS within each processing step.

Finally, the different scores obtained for the five processing steps were then summed up and a global score was obtained (*D*_*TOT*_):

(5) $$\begin{array}{l} D_{TOT}=D_{F1}+D_{F2}+D_{F3}+D_{F4}+D_{F5} \end{array}$$

With this information, the global Performance Hygiene and Safety Score (*PHSS*_*FTOT*_, %) was calculated as:

(6) $$\begin{array}{l} PHSS_{F_{TOT}}=1-\left(\frac{D_{TOT}}{D_{\max F_{TOT}}}\right)x\;100 \end{array}$$

where *D*_*maxF*_*TOT*__ was defined as the maximum score that can be potentially obtained for the five processing steps evaluated.

#### Calculation of weighted performance hygiene and safety scores (PHSS_w_)

To measure the relative importance of each processing step on the final quality and safety of table olives, an Expert Knowledge Elicitation process (EKE) was performed. Expert elicitation is a process for quantifying expert opinion regarding uncertainties to address research problems in areas where traditional scientific research is infeasible or not yet available. Because uncertainties in the probabilistic model can be described in terms of probability distributions EKE can be considered for the derivation of distribution parameters (Clemen and Winkler, 1999). For the present study, the relative importance of each processing step was quantified by a group of 25 quality inspectors from the table olive sector together with scientists and public health authorities. A percentage from 0 to 100% was individually assigned to each processing step (% *F*_*i*_) and a triangular distribution with three parameters; most probable number, minimum and maximum. These percentages (% *F*_*i*_) were included in the model to weight the steps according to the experts' opinion. For the processing step *F*_*i*_, the weighted PHSS values (*PHSS*_*w*_) were calculated as:

(7) $$\begin{array}{l} PHSS_{w}=\left(\frac{D_{\max F_{TOT}}}{D_{TOT}}\right)+\left(\frac{\%F_{i}}{\text{100}}\right)x\left(\frac{PHSS_{F_{i}}}{\text{100}}\right) \end{array}$$

It should be noted that *PHSS*_*Fi*_ values provide a measure of the global variability in the elaboration process while *PHSS*_*w*_ values are indicative of the individual contribution of each processing step to the overall quality and safety of the finished product.

#### Statistical analyses

Boxplots including the main descriptive statistics (mean, standard deviation, 5th, 95th percentiles) were generated for each model output, i.e., *PHSS*_*Fi*_ and *PHSS*_*w*_ values. Descriptive statistics of the final distribution outputs were used to quantify model variability associated to the hygienic-sanitary conditions in each processing step. Uncertainty was considered using the 95% CI for the microbiological results and scores. Further, an ANOVA analysis was also performed to find significant differences between processing steps in relation to the PHSS values calculated (*p* < 0.05).

Further, Spearman correlation coefficients were obtained through a sensitivity analysis to identify the most relevant processing steps, samples and parameters that may exert an influence on the final product quality and safety. To avoid unrealistic results of the model, the Spearman's rank order correlation in the @Risk software was used to assume a previous high-dependence association between microbial loads found in olive fruits and brines (*r* = 0.75). The probabilistic model was run with a MonteCarlo simulation in @Risk v7.5 with 10,000 iterations.

## Results and discussion

### Hygienic-sanitary status of *Aloreña* table olive processing

To evaluate the status of the hygienic-sanitary conditions throughout the *Aloreña* table olive processing, a total of 658 microbiological and physico-chemical data were obtained from brines, olive fruits, air environment, food-contact surfaces, food handlers, and water samples in three industries. Besides, finished packed table olives were characterized after processing just before commercialization. The mean concentrations together with the assigned scores to each processing step are represented in Tables 1–5.

#### Processing step 1: reception of raw materials and fermentation

In Table 1, the status of olive brines and fruits once fermentation was completed indicated a relatively high concentration of MB and Y/M. Olive brines presented a mean value of 6.20 log_10_ cfu/ml of MB while Y/M concentration corresponded to 5.00 log_10_ cfu/ml. LAB concentration was also higher than 6 log_10_ cfu/ml. These microbiological values are in agreement with data reported by Arroyo-López (2007) for this type of table olive specialty in this step. In olive fruits, microbial loads were slightly lower though presence of SRC was detected at low levels (around 1 log_10_ cfu/g). Neither Ent nor CPS were detected in brines or fruits samples in this processing step. The absence of Ent was related with the low pH obtained after fermentation of fruits (Garrido-Fernandez et al., 1997). Air contamination was qualified as intermedium for MB (average count of 2.32 log_10_ cfu/m^3^) while lower values were obtained for Y/M (1.75 log_10_ cfu/m^3^). Regarding physico-chemical data, it should be noted that some deficiencies were denoted regarding pH values of brines, which were slightly higher than 4.3, meaning that these samples would not comply with the requirements stated in the international laws (Codex Alimentarius 1981, rev 2013; IOC, 2004), where maximum allowable pH is 4.3. On the contrary, data obtained for FA and salt can be considered as normal (Garrido-Fernandez et al., 1997; Arroyo-López, 2007). Finally, water samples presented unacceptable values of MB, Col and SRC though these two later groups were detected after samples enrichment.

#### Processing step 2: olive washing and cracking

After fermentation of fruits, olives were washed and cracked by industry. Cracking step is considered as a critical control point in the HACCP system since microbial hazards present in contaminated olive fruits can be spread during the cracking process to non-contaminated fruits, brines or food-contact surfaces. The microbiological quality of brines and fruits was very similar to the processing step 1 (Table 2). However, presence of Ent was observed at high levels in the hopper surfaces (mean = 1.86 log_10_ cfu/cm^2^) and could have been probably transferred to olive brines since more than 4 log_10_ cfu/ml was observed in some of evaluated samples. These loads were also observed by other authors (Arroyo-López, 2007; Alves et al., 2012) ranging from 2.6 to 3.5 log_10_ cfu/ml in brines at the beginning of the fermentation period, but there is no information available on the surface of machinery, containers, operators, etc., in olive industry. The presence of Ent is not desired in table olives because they could jeopardize the stability and safety of finished products (Garrido-Fernandez et al., 1997). Air contamination was classified as “intermediate,” according to the microbial concentrations obtained (100–300 cfu/m^3^).

#### Processing step 3: selection and addition of olive dressings

In this step, samples collected corresponded to air environment, conveyor belts, handlers' gloves, olive fruits, and olive dressings (red pepper, garlic and herbal mixture). Overall, high microbial counts of MB and Ent were obtained in samples from conveyor belts. Presence of Ent was detected in olive fruits (mean = 2.30 log_10_ cfu/g), and in olive dressings, being higher for the herbal mixture (mean = 6.45 log_10_ cfu/g). Addition of herbs and spices to olive fruits could imply an increase in the microbial load of finished products given the high concentrations of MB, Y/M, LAB, and Ent (Arroyo-López, 2007). Besides, product safety could be compromised since high concentrations of CPS were detected in herb samples (mean = 4.42 log_10_ cfu/g) together with the presence of SRC (Table 3). However, the influence of seasoning material in table olive processing has not been studied in detail in spite of their considerable influence on quality and safety of finished products. Samples from food handlers presented relatively low counts of MB. Ent were not detected in handlers' gloves.

#### Processing step 4: packaging process

Table 4 represents the microbial counts obtained in the packaging step. A substantial reduction in mean counts were observed in comparison to the previous steps. This could be attributed to the inhibitory effect of salt concentration and pH together with the renovation of brines which imply a reduction in the microbial load of olive fruits. However, low counts of Ent were observed in fruits (mean = 2.68 log_10_ cfu/g) that could probably be associated to the high concentrations detected in olive dressings and transferred to this step. Olive brines had good microbiological quality as well as water samples. CPS and SRC were not detected in any sample.

#### Processing step 5: finished product

Finished products just before commercialization presented lower microbial concentration of all groups analyzed which means that contamination during processing can be sporadic and product formulation (especially salt, pH values, and addition of preservatives) does not allow microbial growth during shelf life. In Table 5, it can be observed that concentrations were below 4 log_10_ cfu/g in all samples evaluated. Further, Ent, SRC and CPS were not detected in any sample. Absence or low levels of Ent in finished product is in line with data obtained by other authors which reflect their survival in olive packaging only during the first days (Bautista-Gallego et al., 2010; Romero-Gil et al., 2016). LM and Salm were not detected in any sample of fruits and brines in the finished product. This data is in concordance with the study carried out by Medina et al. (2016), who related the inhibition of diverse food-borne pathogens (among them *Listeria* and *Salmonella*) in *Aloreña de Málaga* brines by the presence of diverse phenolic compounds.

It should be noted that for some samples, pH values and salt concentrations exceeded the recommended limits for table olives (pH > 4.3; NaCl < 6%) which could imply that halotolerant or acidic-resistant microorganisms could proliferate during storage if they are previously present in the intermediate fruits and /or brines. Further, mean content of residual sugar in olive fruits was 2.70 g/l, which could support microbial growth. This is particularly relevant for olive production, since yeasts have the capacity to produce refermentation in presence of residual sugars (Loureiro and Malfeito-Ferreira, 2003). In this context, it is highly important to reduce yeasts concentration during the table olive processing in order to improve product stability and shelf-life (Alves et al., 2012). Bautista-Gallego et al. (2013) related yeasts as the main microbial agent causing instability of *Aloreña de Málaga* packaging at salt concentration above 5.0%, while Romero-Gil et al. (2016) point to LAB as spoilage microorganisms when the salt concentration was below this critical level.

### Probabilistic assessment of hygiene and safety of *Aloreña de Málaga* table olives

#### Simulation results of PHSS and PHSS_w_ values

In the present study, a decision-making scoring system was suggested to operationalize hygienic-sanitary conditions in the *Aloreña de Málaga* table olives processing. The degree of fulfillment and the variability in the hygienic and safety conditions was quantified at each processing step (PHSS_Fi_) as well as for the global process through the calculation of PHHS_FTOT_. Besides, the relative importance of processing conditions was quantified by experts' elicitation. This information served to estimate the PHSS_w_ values.

**Figure 2:**
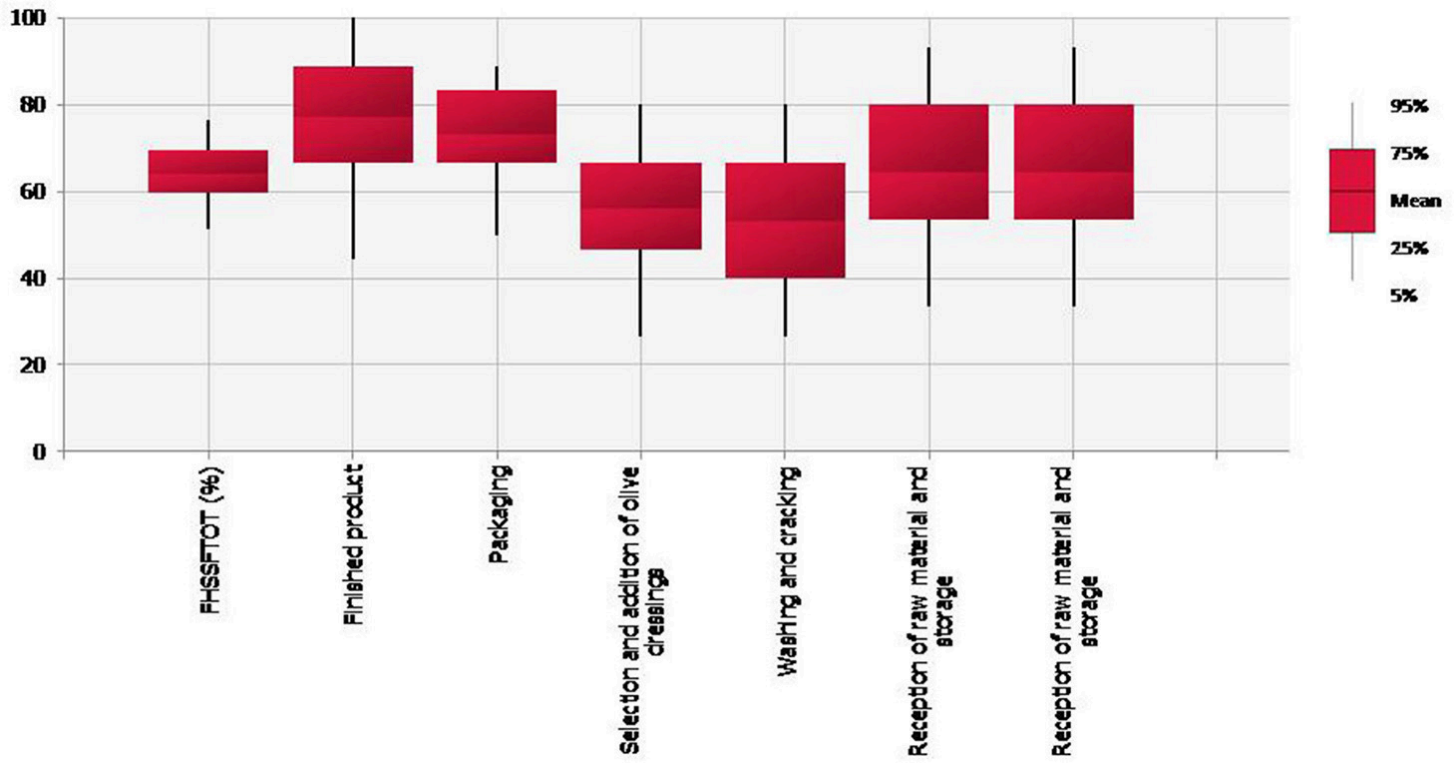
Boxplot representing the mean, 5, 25, 75, and 95th values of the Performance Hygiene and Safety Score at the different processing steps (PHSS_Fi_) together with the global PHSS_FTOT_ (%).

Figure 2 shows the simulation results of PHSS_Fi_ and PHSS_FTOT_ outputs. The mean value of the global Performance Hygiene and Safety Score for the *Aloreña de Málaga* table olives processing (PHHS_FTOT_) was 64.82% (90th CI: 52.78–76.39%) indicating a variation in the hygienic practices in the evaluated processing steps among different industries. Washing and cracking, and selection and addition of olive dressings were detected as the most deficient steps since the lowest PHSS_Fi_ values were obtained (*p* < 0.05) (mean = 53.02 and 56.62% respectively). Especially for washing and cracking, variability in processing conditions among facilities was the highest (90th CI: 26.67–80.00%) and high contamination of brines and fruits were obtained. Packaging and finished products showed higher PHSS_Fi_ values (mean > 73%) probably attributed to product formulation (combination of low pH and high NaCl levels) together with the addition of new brines and preservatives that contributed to a reduction of microbial contamination at the packaging step.

PHHS_w_ values were mainly based on the elicitation scores assigned by different experts from the *Aloreña de Málaga* table olive processing sector. Further, triangular distributions with minimum, most probable and maximum scores for each processing step were adjusted. Distributions were used as modeling inputs to estimate the individual contribution of the processing steps to the overall hygienic-sanitary conditions of finished products. In Table 7, descriptive statistics and percentiles of simulated distributions indicated that the finished product was assigned by experts as the most relevant step from a hygiene and safety point of view, having the highest 95th percentile (36.22%) followed by the selection and addition of olive dressings (30.84%). In contrast, reception of raw materials and fermentation, olive washing and cracking and packaging steps had the lowest 95th percentiles (24.49–27.64%). The main premise behind an expert elicitation method is that the method employed incorporates the knowledge and experience of the experts, and reduces the judgment biases. In the present study, the use of questionnaires allowed to collect information from quality inspectors of the table olive sector. Expert opinions can be used to address important questions and uncertainties in risk analysis. However, one of the limitations of expert elicitation is that sometimes experts may not describe accurately their actual knowledge so that data selection should be taken with caution.

**Table 7:** Elicitation scores (%) assigned by individual experts (*n* = 25) from the *Aloreña de Málaga* table olive sector.

**Processing step**	**Distribution**	**Mean**	**S.D**.	**5th Perc**	**95th Perc**
Reception of raw material and fermentation	RiskTriang(5;10;40)	16.49	6.18	8.02	27.64
Olive washing and cracking	RiskTriang(5;20;30)	17.10	4.61	9.30	24.49
Selection and addition of olive dressings	RiskTriang(10;20;40)	21.46	5.29	13.49	30.84
Packaging process	RiskTriang(10;20;30)	18.86	3.92	12.66	25.61
Finished product	RiskTriang(5;40;40)	26.12	6.74	13.79	36.22

*Parameters used for triangular distributions are shown together with simulated statistics*.

**Figure 3:**
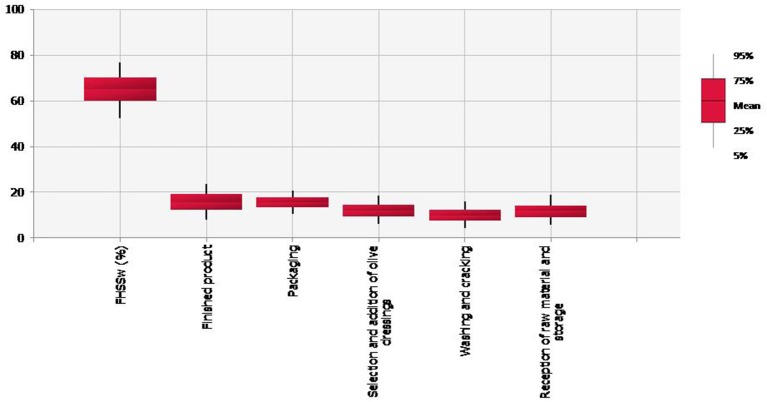
Boxplot representing the mean, 5, 25, 75, and 95th values of the individual contribution of the processing steps on the weighted Performance Hygiene and Safety Score PHSS_w_ (%).

In Figure 3, the relative contribution of each processing step on the PHSS_w_ was represented, according to the values provided by the experts (Table 7). Significant differences in PHSS_w_ values were obtained between packaging process and finished products, and the remaining processing steps (*p* < 0.05). The mean value of PHSS_w_ was 65.53% (90th CI: 53.12–77.52%), very similar with PHSS_FTOT_ with 64.82%. As above mentioned for FHSS_FTOT_, the final processing steps obtained higher values for PHSS_w_ being the finished product the most relevant one (mean = 18.44%; 90% CI: 10.34–25.33%). However, it should be noted that PHSS_w_ values are influenced by the weighting percentage assigned by the experts. In this case, the final steps were considered highly relevant for preserving the stability of the finished product and its shelf-life extension.

#### Sensitivity analysis of PHSS and PHSS_w_ values on the type of sample and processing step

**Figure 4:**
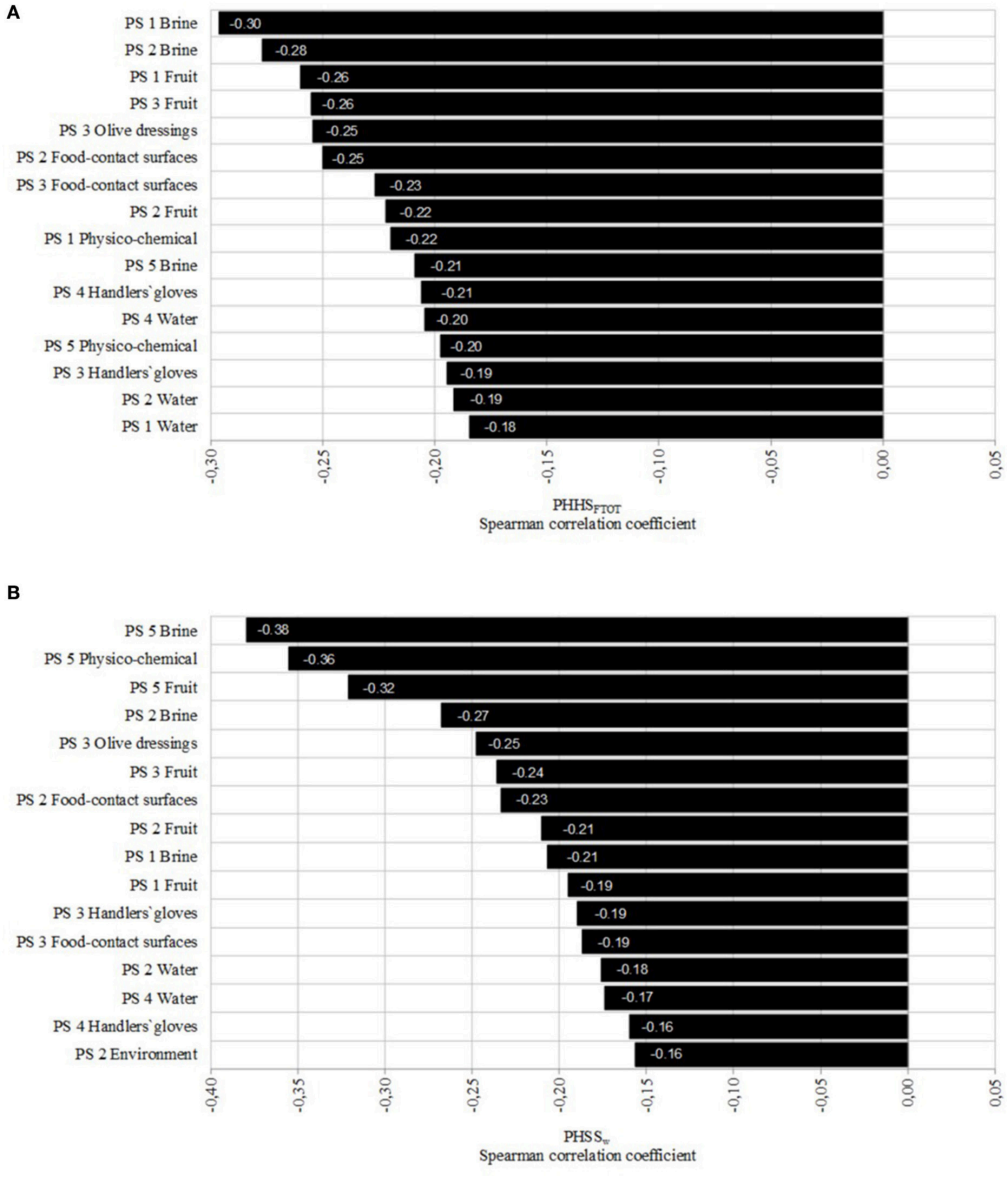
Spearman correlation coefficients describing the relative influence of the type of sample on the final global Performance Hygiene and Safety Score (PHSS_FTOT_) **(A)** and the weighted Performance Hygiene and Safety Score (PHSS_w_) **(B)**. PS stands for the processing step.

In Figure 4, Spearman correlation coefficients describing the relative influence of the type of sample on the PHSS_FTOT_ and on the PHSS_w_ are represented. As PHSS_FTOT_ were calculated without weighting the processing steps (all of them were considered equally relevant for final product quality and safety), correlation coefficients were higher for the primary steps which corresponded to the most contaminated samples. Particularly, the microbiological quality and safety of used brines presented a high correlation (−0.30 for brines used during the reception of raw materials and storage; and −0.28 for brines used during washing and cracking of table olives) with the final PHSS_FTOT_ values, followed by results obtained in processing step 3 (selection and addition of olive dressings) for intermediate fruits and olive dressings.

On the contrary, for PHSS_w_ values, (Figure 4B) the finished product presented the highest correlations (fruits, brines and physico-chemical values) since this step contributed mostly to the increase of PHSS_w_.

**Figure 5:**
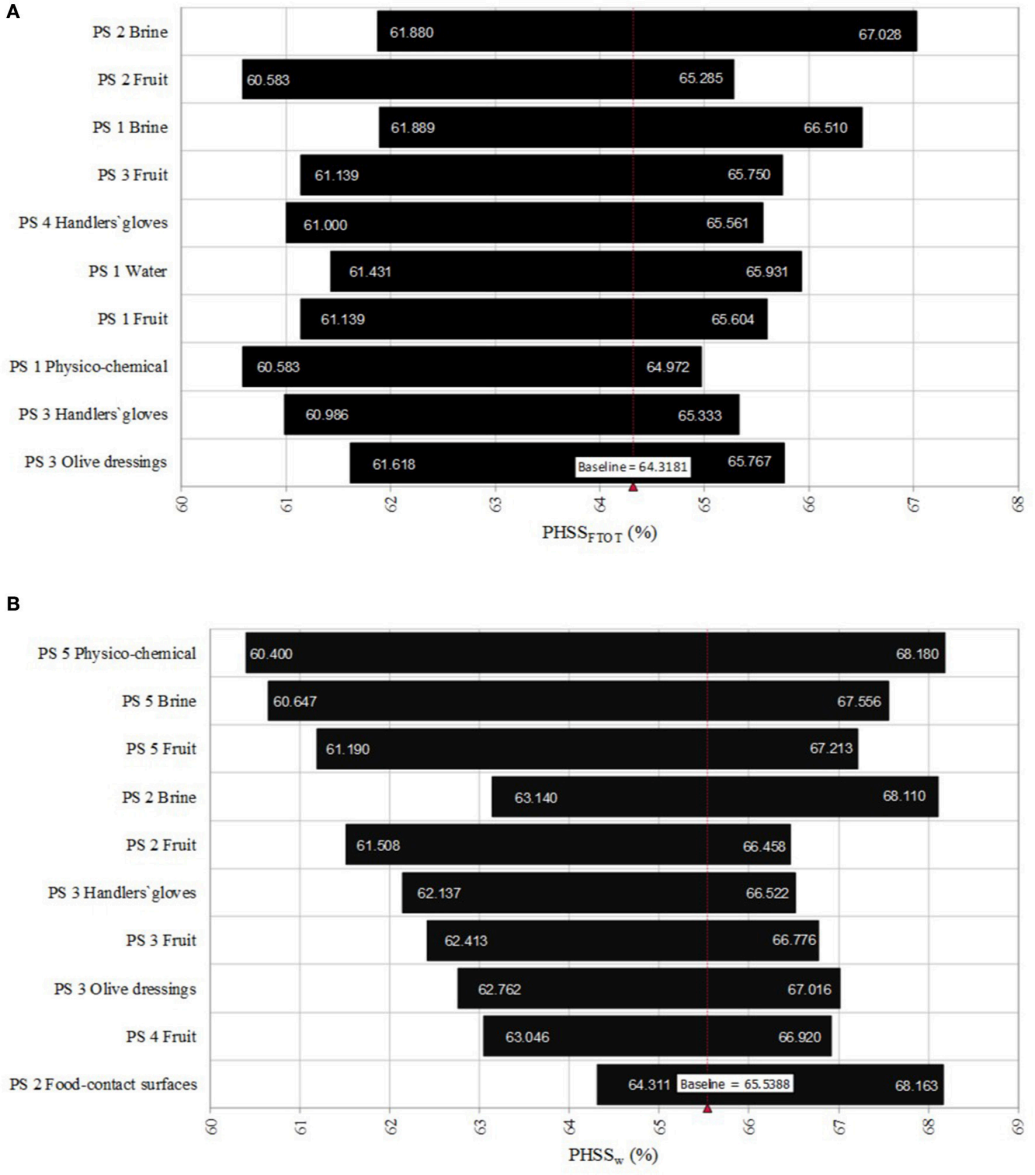
Results of the sensitivity analysis describing the relative influence of the type of sample on the variation of the mean value for the global Performance Hygiene and Safety Score (PHSS_FTOT_) **(A)** and the weighted Performance Hygiene and Safety Score (PHSS_w_) **(B)**. PS stands for the processing step.

Sensitivity analysis was also performed on the relative variation of each type of sample on the mean PHSS_FTOT_ and PHSS_w_ values (Figure 5). It can be concluded that intervention measures focused on reducing the contamination of washing brines (processing step 2) could lead to an improvement of PHSS_FTOT_ value to 67.03 %. On the contrary, contamination of fruits during washing and cracking could also lead to a reduction of PHSS_FTOT_ values to 60.58%.

Regarding PHSS_w_, in Figure 5B, physico-chemical values and contamination of brines and fruits in the processing step 5 (finished product) produced the widest variation of PHSS_w_ values. However, as seen in Table 5, contamination of brines and fruits were relatively lower than in previous steps, being influenced by the addition of olive dressings as well as by product formulation. It should be remarked that corrective measures implemented during washing and cracking can be equally effective on the PHSS_w_ (68.11%). It was also identified that cleaning of washing hoppers at processing step 2 could increase the final PHSS_w_ value up to 68.16%.

**Figure 6:**
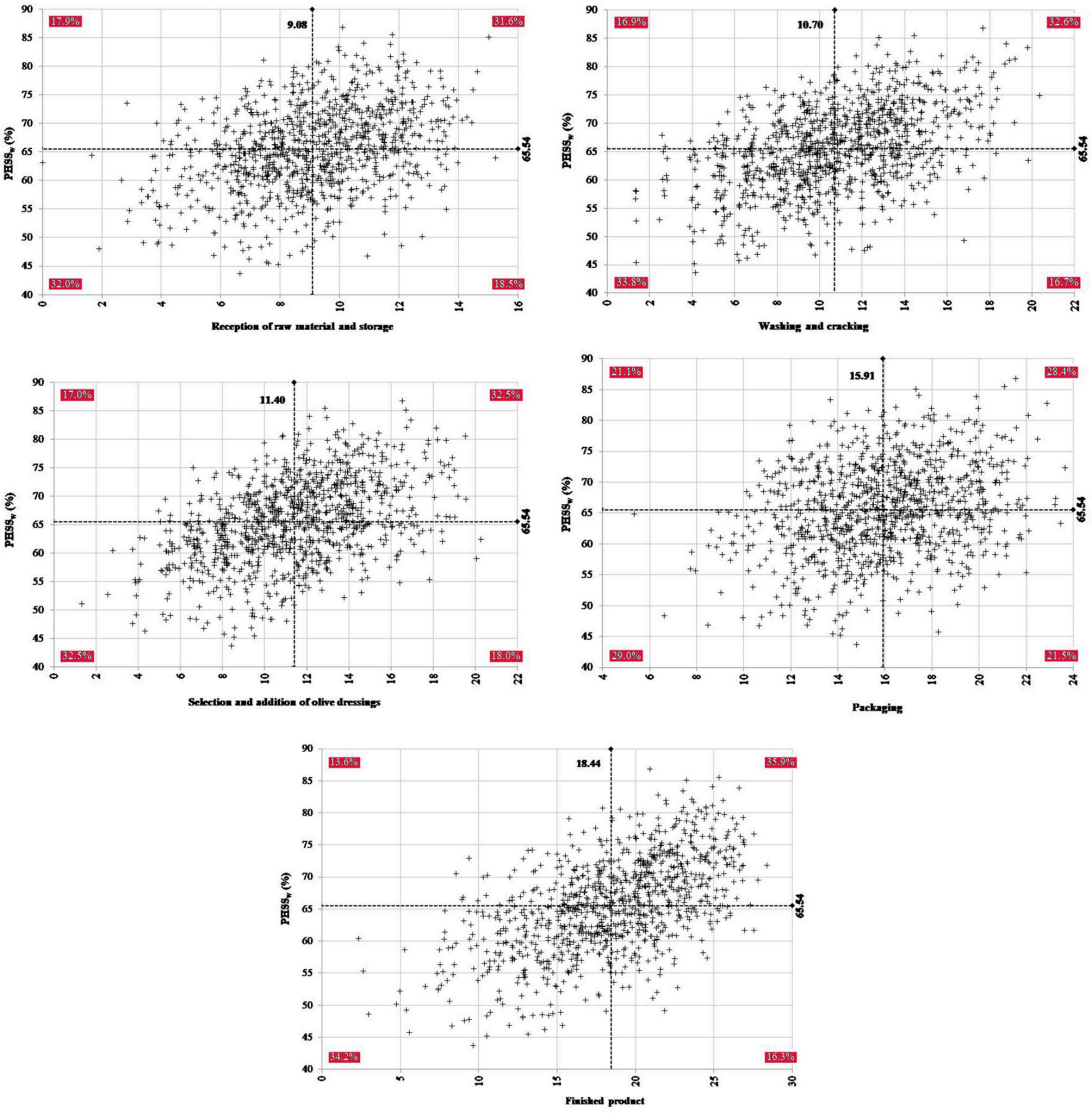
Relationship between simulated PHSS_w_ values and individual contributions of the evaluated processing steps.

In Figure 6, a direct correlation was found between simulated PHSS_w_ values and relative contributions of each processing step. Simulated results showed that the proportion of directly correlated values was higher for steps 2 (66.4%) and 5 (70.1%). Packaging was identified as the step with lesser proportion of directly correlated values with PHSS_w_ (57.4%).

To date, there are not probabilistic tools based on the application of FQSMS in the table olive sector. There are other tools in literature in which a systematic analysis of microbial counts was used to assess the degree of performance of a FQSMS (Jacxsens et al., 2009; Lahou et al., 2014). These approaches are based on a selection of critical sampling location, selection of microbial parameters, assessment of sampling frequency, selection of sampling and analytical methods and data processing and interpretation. Different microbial safety levels are assigned according to the compliance with legal criteria for both microbial hygiene and safety. The approach followed in the present study is in agreement with the principle behind Microbial Assessment Schemes (MAS) in which low concentration of microorganisms and small variability indicate an effective FQSMS (Sampers et al., 2010; Luning et al., 2011).

In conclusion, it is suggested that corrective measures should be focused on reducing the microbial contamination of brines and fruits at primary steps, together with the implementation of novel treatments on olive dressings (irradiation, scalding, ozonization, etc.) to reduce their microbial load since contamination can persist in brines and fruits during table olive processing. According to the suggested approach, these preventive measures can be equally or even more effective than modifying product formulation to lower pH values and higher salt concentrations. In addition, industry could reduce the levels of salt and preservatives in packaging producing a healthier product. The results presented are currently integrated within a software tool which will provide stakeholders with an easy-to-use, flexible and useful probabilistic decision-making scoring system for the *Aloreña de Málaga* table olive food sector. Furthermore, the approach can be extended to other olive varieties and elaboration methods including alternative treatments and steps as long as the information about scores weighing becomes available.

## Author contributions

MR, FR-G, EM, VR, AP, and GP-I executed the experimental work and microbiological and physico-chemical analysis. FA-L, FR, RG-G, and AV planning the experiment and written the manuscript.

### Conflict of interest statement

The authors declare that the research was conducted in the absence of any commercial or financial relationships that could be construed as a potential conflict of interest.

## References

[B1] Al Dagal M. Mo O. Fung D. Y. C. Kastner C. (1992). A case study of the influence of microbial quality of air on product shelf life in a meat processing plant. Dairy Food Environ. Sanita 12, 69–70.

[B2] Alves M. Gonçalves T. Quintas C. (2012). Microbial quality and yeast population dynamics in cracked green table olives' fermentations. Food Control 23, 363–368. 10.1016/j.foodcont.2011.07.033

[B3] Arroyo-López F. N. (2007). Doctoral Thesis: Conservación y Envasado de Aceitunas de Mesa “Ali-adas” de la Variedad Manzanilla-Alore-a. Dise-o de Modelos Matemáticos Para el Crecimiento e Inhibición de Microorganismos. Departamento de Cristalografía, Mineralogía y Química Agrícola. Universidad de Sevilla.

[B4] Arroyo-López F. N. Bautista-Gallego J. Romero-Gil V. María Baquero J. M. García-García P. Jiménez-Díaz R. (2012). Fermentation of olive fruit, in Handbook of Plant-Based Fermented Food and Beverage Technology, ed Hui Y. H. (Boca Ratón, FL: CRC Press, Taylor and Francis Group), 307–327.

[B5] Arroyo-López F. N. García-García P. Rodríguez-Gómez F. Garrido-Fernández A. (2016). Olives: types and consumption, in The Encyclopaedia of Food and Health, vol. 4, eds Caballero B. Finglas P. Toldrá F. (Oxford: Academic Press), 167–170.

[B6] Bautista-Gallego J. Arroyo-López F. N. Durán-Quintana M. C. Garrido-Fernández A. (2010). Fermentation profiles of Manzanilla-Alore-a cracked green table olives in different chloride salt mixtures. Food Microbiol. 27, 403–412. 10.1016/j.fm.2009.11.015 20227606

[B7] Bautista-Gallego J. Arroyo-López F. N. Romero-Gil V. Rodríguez-Gómez F. García-García P. Garrido-Fernández A. (2013). Microbial stability and quality of seasoned cracked green Alore-a table olives packed in diverse chloride salt mixtures. J. Food Prot. 76, 1923–1932. 10.4315/0362-028X.JFP-12-504 24215697

[B8] Clemen R. T. Winkler R. L. (1999). Combining probability distributions from experts in risk analysis. Risk Anal. 19, 187–203. 10.1111/j.1539-6924.1999.tb00399.x 10859775

[B9] Codex Alimentarius Commission (1969a). Code of Hygienic Practice for Canned Fruit and Vegetable Products. CAC/RCP 2-1969.

[B10] Codex Alimentarius Commission (1969b). General Principles of Food Hygiene. CAC/RCP 1-1969 (Review 1997 and 2003).

[B11] Codex Alimentarius Commission (1979). Code of Hygienic Practice for Low and Acidified Low Acid Canned Foods. CAC/RCP 23-1979.

[B12] Codex Alimentarius Commission (1997). Principles for the Establishment and Application of Microbiological Criteria for Foods. CAC/GL 21–1997.

[B13] Codex Alimentarius Commission (1981). Codex Standards for Table Olives. CODEX STAN 66-1981 (Review 1987 and 2013).

[B14] Commission Regulation (2007). Commission Regulation (EC) No 1441/2007 of 5 December 2007 amending Regulation (EC) No 2073/2005 on microbiological criteria for foodstuffs. Official J. Eur. Union L322, 12–29. Available online at: http://data.europa.eu/eli/reg/2007/1441/oj

[B15] Commission Regulation (2002). Commission Regulation (EC) No 178/2002 of the European parliament and of the council of 28 January 2002 laying down the general principles and requirements of food law, establishing the European Food Safety Authority and laying down procedures in matters of food safety. Official J. Eur. Union L31, 1–24. Available online at: http://data.europa.eu/eli/reg/2002/178/oj

[B16] Commission Regulation (2004). Commission Regulation (EC) No 852/2004 of the European parliament and of the council of 29 April 2004 on the hygiene of foodstuffs. Official J. Eur. Union, L226, 3–21. Available online at: http://data.europa.eu/eli/reg/2004/852/2009-04-20

[B17] Federation des Industries Condimentaires de France (2000). Code des pratiques loyales pour les olives de table.

[B18] Garrido-Fernandez A. Fernandez-Dıez M. J. Adams R. (1997). Table Olives. Production and Processing. London: Chapman and Hall.

[B19] Grover A. K. Chopra S. Mosher G. A. (2016). Food safety modernization act: a quality management approach to identify and prioritize factors affecting adoption of preventive controls among small food facilities. Food Control 66, 241–249. 10.1016/j.foodcont.2016.02.001

[B20] IOC (2004). Trade Standard Applying to Table Olives. COI/ NC n°1. Madrid: International Olive Council.

[B21] IOOC (2017). World Table Olive Figures. International Olive Oil Council. Available online at: http://www.internationaloliveoil.org/estaticos/view/132-world-table-olive-figures (Accessed April 18, 2017).

[B22] ISO (2002). ISO Microbiology of Food and Animal Feeding Stuffs – Horizontal Method for the Detection of Salmonella sp. ISO 6579: 2002.

[B23] ISO (2017). ISO Microbiology of Food and Animal Feeding Stuffs – Horizontal Method for the Detection and Enumeration of Listeria monocytogenes – Part 1: Detection Method. ISO 11290-1:1996/Amd1:2017.

[B24] ISO (2004). ISO Microbiology of Food and Animal Feeding Stuffs – Horizontal Method for the Detection and Enumeration of Listeria monocytogenes – Part 2: Enumeration Method. ISO 11290-2:1998/Amd1:2004

[B25] ISO (1999). ISO Microbiology of Food and Animal Feeding Stuffs – Horizontal Method for the Enumeration of Coagulase-positive Staphylococci (Staphylococcus aureus and Other Species) – Part 2: Technique Using Rabbit Plasma Fibrinogen Agar Medium. ISO 6888-2:1999.

[B26] Jacxsens L. Kussaga J. Luning P. A. Van der Spiegel M. Devlieghere F. Uyttendaele M. (2009). A microbial assessment scheme to measure microbial performance of Food Safety Management Systems. Int. J. Food Microbiol. 134, 113–125. 10.1016/j.ijfoodmicro.2009.02.018 19327860

[B27] Lahou E. Jacxsens L. Van Landeghem F. Uyttendaele M. (2014). Microbiological sampling plan based on risk classification to verify supplier selection and production of served meals in food service operation. Food Microbiol. 41, 60–75. 10.1016/j.fm.2014.01.012 24750814

[B28] López-López A. Garrido-Fernández A. (2006). Producción, Elaboración, Composición y Valor Nutricional de la Aceituna Alore-a de Málaga. Málaga: Redagua, S.L., Pizarra.

[B29] Loureiro V. Malfeito-Ferreira M. (2003). Spoilage yeasts in the wine industry. Int. J. Food Microbiol. 86, 23–50. 10.1016/S0168-1605(03)00246-0 12892920

[B30] Luning P. A. Jacxsens L. Rovira J. Osés S. M. Uyttendaele M. Marcelis W. J. (2011). A concurrent diagnosis of microbiological food safety output and food safety management system performance: cases from meat processing industries. Food Control 22, 555–565. 10.1016/j.foodcont.2010.10.003

[B31] Medina E. Romero-Gil V. Garrido-Fernández A. Arroyo-López F. N. (2016). Survival of foodborne pathogens in natural cracked green olives. Food Microbiol. 59, 104–111. 10.1016/j.fm.2016.05.017 27375250

[B32] Nanyunja J. Jacxsens L. Kirezieva K. Kaaya A. N. Uyttendaele M. Luning P. A. (2015). Assessing the status of food safety management systems for fresh produce production in East Africa: evidence from certified green bean farms in Kenya and noncertified hot pepper farms in Uganda. J. Food Prot. 78, 1081–1089. 10.4315/0362-028X.JFP-14-364 26038896

[B33] Ren S. Ni X. Xu H. Den J. Wang Y. Sun J.. (2010). The relationship between hand area and hand contamination. Am. J. Infect. Control 39, 66–68. 10.1016/j.ajic.2010.06.014 21074895

[B34] Ren Y. He Z. Luning P. A. (2016). A systematic assessment of quality assurance-based food safety management system of Chinese edible oil manufacturer in view of context characteristics. Total Qual. Man. 27, 897–911. 10.1080/14783363.2016.1187995

[B35] Romero-Gil V. Rodríguez-Gómez F. Garrido-Fernández A. García-García P. Arroyo-López F. N. (2016). *Lactobacillus pentosus* is the dominant species in spoilt packaged Alore-a de Málaga table olives. LWT Food Sci. Technol. 70, 252–260. 10.1016/j.lwt.2016.02.058

[B36] Royal Decree (2001). Royal Decree 1230/2001: Technical and Sanitary Regulation Concerning the. Processing, Circulation and Sale of Table Olives.

[B37] Royal Decree (2003). Royal Decree 140/2003, of 7th February, Approving the Sanitary Quality Criteria of Water for Human Consumption.

[B38] Sampers I. Jacxsens L. Luning P. A. Marcelis W. Dumoulin A. Uyttendaele M. (2010). Relation between Campylobacter contamination and performance of Food Safety Management Systems in poultry meat industries. J. Food Protect. 73, 1447–1457. 10.4315/0362-028X-73.8.1447 20819354

[B39] Sánchez A. H. De Castro A. Rejano L. Montaño A. (2000). Comparative study on chemical changes in olive juice and brine during green olive fermentation. J. Agr. Food Chem. 48, 5975–5980 10.1021/jf000563u 11141267

[B40] Sneed J. Strohbehn C. Gilmore S. A. Mendonça A. (2004). Microbiological evaluation of foodservice contact surfaces in Iowa assisted-living facilities. J. Am. Diet. Assoc. 104, 1722–1724. 10.1016/j.jada.2004.08.026 15499361

[B41] Stadlmüller L. Matt M. Peter Stüger H. Komericki-Strimitzer T. Jebousek K. Luttenfeldner M. (2017). An operational hygiene inspection scoring system for Austrian high-risk companies producing food of animal origin. Food Control 77, 121–130. 10.1016/j.foodcont.2017.01.019

[B42] Tzamalis P. Panagiotakos D. Drosinos E. (2016). A 'best practice score' for the assessment of food quality and safety management systems in fresh-cut produce sector. Food Control 63, 179–186. 10.1016/j.foodcont.2015.11.011

[B43] Valero A. Medina E. Arroyo-López F. N. (2017). Microbial hazards and their implications in the production of table olives, in Foodborne Pathogens and Antibiotic Resistance, ed Singh O. V. (Hoboken, NJ: Willey and Sons Inc.), 119–138.

